# Comparative histomorphometric and transcriptomic analysis reveals potential genetic determinants of pelage variation between hairy and coarse-woolly sheep

**DOI:** 10.1186/s12864-025-12260-y

**Published:** 2025-11-14

**Authors:** Qing Liang, Dejun Ji, Xianwei Wang, Xu Peng, Jingxin Zhang, Chenjuan Ge, Yulu Zheng, Tengyun Gao, Yinghua Shi, Zejun Xu, Tong Fu, Sen Ma

**Affiliations:** 1 College of Animal Science and Technology, Henan Agricultural University, Zhengzhou, 450002 Henan China; 2 College of Animal Science and Technology, Yangzhou University, Yangzhou, 225000 Jiangsu China; 3 Henan Provincial Animal Husbandry General Station, Zhengzhou, 450002 Henan China

**Keywords:** Hairy sheep, Coarse-woolly sheep, Pelage phenotype divergence, Subcutaneous adiposity, Immunoregulation, Fiber traits

## Abstract

**Background:**

The unique pelage composition and developmental pattern endow hairy sheep with superior heat tolerance and minimized wool-shearing needs, compared with coarse-woolly sheep. However, the genetic mechanisms underlying pelage differentiation between these two sheep types remain incompletely understood to date, thereby limiting the development of molecular breeding strategies for cultivating climate-resilient and cost-effective sheep breeds in climate-changing era.

**Results:**

Through integrated histomorphometric and transcriptomic analysis of Australian White Sheep (AWS, hairy phenotype) and Hu Sheep (HS, coarse-woolly phenotype), we identified three distinct evolutionarily conserved fiber types in AWS (heavily medullated kemp, medullated crimp hair, and non-medullated wool). In contrast, HS exhibited a bimodal fiber distribution without kemp fibers. Compared with AWS, HS had significantly longer hair fibers and markedly reduced subcutaneous adiposity. Transcriptomic profiling identified 370 differentially expressed genes (DEGs): genes enriched in AWS (e.g., *DGAT2L6*, *FOXO6*, *CIDEA*, *ADIG*) were clustered in lipid metabolism pathways, while genes upregulated in HS (e.g., *CSN2*, *LOC101102413*, *CSF3R*, *CXCR2*) were significantly associated with immunomodulatory functions. Additionally, hair intermediate filament- and matrix-associated candidate genes (e.g., *LOC114113348*/*KAP20-2*, *LOC101111178*/*KRT6A*) and *EDA2R* exhibited breed-specific expression patterns, which were respectively linked to differences in fiber curvature and length.

**Conclusion:**

Our integrated analysis identifies subcutaneous adiposity and immunoregulation as novel potential key modulators of pelage phenotype divergence between hairy and coarse-woolly sheep—with candidate genes (e.g., *KAP20-2*, *KRT6A*, *EDA2R*) linked to breed-specific fiber traits (e.g., curvature, length). The identified genetic signatures further offer potential actionable targets for precision breeding of climate-resilient and cost-effective sheep, addressing the gap in molecular strategies for pelage-related traits improvement.

**Supplementary Information:**

The online version contains supplementary material available at 10.1186/s12864-025-12260-y.

## Introduction

Sheep (*Ovis aries*) are among the earliest domesticated livestock species, with key phenotypes (e.g., horn type, pelage, tail morphology) that have undergone distinct and substantial divergence from their wild ancestors under artificial selection [1]. Historically, ovine hair fibers were critical to human survival, which in turn drove extensive evolution of sheep coat traits (i.e., fiber type, fineness, length, and growth pattern) to adapt to diverse environments and human needs during long-term selection [2, 3]. Modern domestic sheep are generally categorized into two groups based on pelage characteristics: hairy sheep and woolly sheep, with the latter further subdivided into coarse-, medium-, and fine-woolly subtypes [4]. Relative to woolly sheep, hairy sheep possess ancestral-type pelage comprising medullated kemp fibers (the coarsest component, with high medullation), medullated hair, and non-medullated wool (the finest component); among these, hair and wool exhibit seasonal growth and shedding, most prominently in spring. In contrast, woolly sheep have pelage consisting of medullated/non-medullated hair and non-medullated wool, and lack seasonal shedding [5]. These pelage differences confer superior heat tolerance to hairy sheep—an attribute that facilitates their adaptation to tropical and subtropical climates and is evidenced by their lower respiratory rates relative to woolly sheep under heat stress [6, 7]. Furthermore, seasonal molting in hairy sheep reduces shearing costs—a benefit that is especially prominent amid the current wool market downturn [8]. However, the mechanisms governing pelage composition and developmental differences between hairy and woolly sheep remain poorly understood, which has impeded the application of modern biotechnological breeding techniques for developing novel sheep varieties with enhanced heat tolerance and auto-molting capabilities, particularly amid ongoing global warming.

Numerous past studies have identified genes and genetic mutations associated with sheep coat traits, although these researches primarily centered on woolly sheep [9]. For instance, several single nucleotide polymorphism (SNP) loci associated with the fibroblast growth factor 5 (*FGF5*) have been found to be closely linked to wool staple length [10], and loss of function of the gene results in increased wool length [11]. Additionally, a suite of genes encoding key hair structural proteins, including keratin (*KRT*) [12, 13], keratin-associated protein (*KAP*) [14], desmoglein 4 (*DSG4*) [15, 16], and trichohyalin (*TCHH*) [17], have been shown to be closely associated with wool fineness or crimp. In contrast, although a small number of studies have identified genes (e.g., *IRF2BP2* [18], *FOXQ1* [4], and *SOX18* [19]) as potential key candidates regulating the hairy phenotype via comparative genomics approaches—with the regulatory mechanism of *IRF2BP2* further validated [20]—research on the coat phenotype of hairy sheep remains relatively scarce.

Hair follicles (HFs), the source of hair fibers, are embedded within skin, a complex local microenvironment that comprises multiple cell types, including immune cells, dermal fibroblasts, and adipocytes [21]. The development of HFs and the consequent hair phenotypes are tightly regulated by these skin-resident cell types. For instance, local subcutaneous adipose tissue has been found to exert complex regulatory effects on HF development [22]. Moreover, several studies have demonstrated that the thickness of subcutaneous adipose tissue in hairy sheep is significantly greater than that in woolly sheep [23, 24]. However, whether subcutaneous adipose tissue influences coat phenotypes in hairy versus woolly sheep remains unclear.

In China, most indigenous sheep breeds are coarse-woolly, with Hu sheep (HS) standing out as a highly valued breed due to its high fecundity. In recent years, elite hairy sheep breeds—including Dorper sheep and Australian White sheep (AWS)—have been introduced to facilitate the genetic enhancement of these indigenous coarse-woolly breeds. Accordingly, using AWS and HS as research models, we conducted integrated analyses (comparative morphological and transcriptomic analyses) to identify key candidate genes and their associated regulatory pathways that potentially mediate coat phenotypic variation. From these analyses, we identified promising candidate genes (e.g., *KAP20-2*, *KRT71*, *EDA2R*) and relevant regulatory pathways (lipid metabolism, immune response). Our findings provide novel insights into the genetic basis of ovine coat diversity and lay a foundation for developing molecular markers to accelerate the breeding of climate-resilient (heat-tolerant, auto-molting) sheep breeds.

## Materials and methods

### Ethical compliance

All experimental procedures strictly adhered to the ARRIVE guidelines 2.0 for animal research and were approved by the Institutional Animal Care and Use Committee (IACUC) of Henan Agricultural University (Protocol No.: HENAU-2018-039). Prior to the initiation of the study, written informed consent was obtained from the owner of the commercial farm, including confirmation of animal ownership and permission for sample collection.

### Collection of skin tissues and hair fiber samples

Five healthy ewes aged 2–3 years from each breed - Australian White sheep (AWS, hairy phenotype) and Hu sheep (HS, coarse-woolly phenotype) - were selected during April 2023 from a commercial farm in Zhongmou County, Henan Province (34°26’N, 114°03’E). Animals were maintained under standardized nutritional conditions with free access to water and TMR-based diet.

Representative photographic documentation of animal phenotypes and fleece characteristics was acquired using an iPhone 15 Plus (Apple Inc., USA) under standardized lighting conditions (5500 K color temperature). Fleece samples (~ 50 g) were collected from the dorsolateral trunk region using sterile surgical shears, ensuring preservation of intact fiber morphology. Prior to tissue excision, the surgical site underwent sequential sterilization with 0.5% iodophor antiseptic solution (5-minute contact time) followed by 75% ethanol swabbing. Local anesthesia was implemented via subcutaneous injection of 0.5 mL 1% procaine hydrochloride (Huamu Animal Health, China; Lot No. 20230315). Full-thickness skin biopsies (ཞ1 cm²) were surgically excised using sterile scalpel, followed by dual rinses in ice-cold phosphate-buffered saline (PBS, pH 7.4) to eliminate hematologic contaminants. Tissue aliquots were processed as follows: (1) molecular analysis: flash-frozen in liquid nitrogen (≤ 30 s exposure) and stored at −80 °C, and (2) histology: fixed in 4% paraformaldehyde for 24 h at 4 °C prior to paraffin embedding.

### Measurement of straightened hair fiber length

Prior to straightened length measurement, fleece samples were manually separated into three subtypes—kemp, guard hair, and wool—based on medullation pattern and fiber diameter, following standardized classification criteria (detailed in Table 1). For each fiber subtype, 10 intact fibers were selected per animal (*n* = 5/breed) for analysis under controlled conditions (20 ± 1℃, 50 ± 5% RH). Fibers were equilibrated in this environment for 24 h to minimize measurement bias. All operations followed Chinese National Standard GB/T 4998 − 1997 (Method for Determination of Wool Fiber Length). Each selected fiber was placed on black velvet cloth, and the operator gently pressed a glass slide onto the fiber with the left hand while aligning one end of the slide with the fiber to set a fixed starting point. A measuring ruler (1 mm precision) was positioned parallel to the fiber. Fine-tipped tweezers were used to gently pull the fiber’s free end until all visible curvature was eliminated (with the fiber lying flat without tension-induced stretching), and the distance from the slide’s aligned end to the fiber’s tip was recorded as its straight length. In addition, high-resolution macro photography was conducted for individual separated hair fibers, with the fibers placed on black velvet cloth. The photography was performed using a Canon EOS RP camera equipped with an EF 100 mm f/2.8 L Macro IS lens, and images were captured under D65 standardized illumination provided by an X-Rite SpectraLight III.

**Table 1:** Classification standards of kemp, hair, and wool

Type	Main characteristics
kemp	Straight, short, thick, brittle, scratchy, and heavily medullated
hair	Crimped, long, softer, finer, and medullated
wool	Most curved, long, softest, finest, and non-medullated

### Light microscopy​

Hair samples were mounted onto glass slides using neutral resin, with manipulation performed via sterile fine-tipped forceps to ensure fiber alignment and avoid contamination. Coverslips were gently lowered onto the mounted samples from one side (rather than directly pressed) to prevent air bubble entrapment. Medullary structure of the hair samples was observed and analyzed using a Motic BA600 bright-field microscope (Motic China Group Co., Ltd., Xiamen, China). Observations were conducted under 400× magnification, and representative images of the medullary architecture were captured using the microscope’s integrated digital camera (Model: Moticam 580) for subsequent morphological analysis.

### Histological section Preparation and staining​

Briefly speaking, skin specimens (1 cm^2^ full-thickness biopsies) were first fixed in 4% paraformaldehyde (pH 7.2; Beyotime Biotechnology, Shanghai, China; Cat No.: P0099) at 4 ℃ for 24 h to preserve tissue morphology. After fixation, specimens underwent gradient dehydration to remove water using an ethanol series: 70% ethanol (1 h), 80% ethanol (1 h), 90% ethanol (1 h), 100% ethanol (2×, 1 h each). Dehydrated tissues were then cleared in xylene (2×, 1 h each) to eliminate ethanol and facilitate paraffin penetration, followed by infiltration with molten paraffin (Paraplast Plus, Sigma-Aldrich, St. Louis, MO, USA) at 60 ℃ for 4 h. Infiltrated tissues were embedded in fresh paraffin to form paraffin blocks, which were sectioned into 5 μm-thick slices using a rotary microtome (Beyotime Biotechnology; Model: RM2235). Sections were mounted onto poly-L-lysine-coated glass slides (Beyotime Biotechnology; Cat No.: S2100) to prevent detachment during staining, and dried at 60 ℃ for 2 h to enhance adhesion.

Transverse and longitudinal sections underwent H&E staining following established protocols [25]. Images were captured at 100× magnification (40× objective lens with numerical aperture 0.75; 10× eyepiece) using a Motic BA600 bright-field microscope (Motic China Group Co., Ltd., Xiamen, China) equipped with a Moticam 580 digital camera—this magnification was selected to ensure clear visualization of skin layer boundaries (epidermis, dermis, hypodermis) for subsequent morphometric analysis. Morphometric parameters were quantified via ImageJ software (v1.54e, National Institutes of Health, Bethesda, MD, USA) with standardized measurement criteria: (1) Epidermal thickness: average distance from the outermost stratum corneum to the dermal-epidermal junction; (2) Dermal thickness: distance from the dermal-epidermal junction to the dermis-hypodermis junction; (3) Hypodermal thickness: average thickness of the subcutaneous adipose tissue layer. For each skin specimen (*n* = 5 animals per breed), 5 non-overlapping fields of view were analyzed per section; all measurements were performed in technical triplicate (repeated 3 times for the same field of view) to minimize random measurement error, with final results expressed as mean ± standard deviation (SD).

### Transcriptomic profile analysis

Total RNA was isolated from 200 mg dorsal skin tissues (*n* = 3 biological replicates per breed) using TRIzol reagent (Invitrogen) following manufacturer protocols. RNA integrity was verified using Agilent 2100 Bioanalyzer (RIN > 8.0), with quantification performed via Qubit 3.0 Fluorometer (Thermo Fisher Scientific). Strand-specific sequencing libraries were prepared using the VAHTS^®^ Universal V8 RNA-seq Library Prep Kit (Vazyme), incorporating poly(A)^+^ mRNA enrichment through Oligo(dT) bead selection. Libraries were size-selected for 300–500 bp inserts and sequenced on DNBSEQ-T7 platforms (MGI Tech) in 150 bp paired-end mode.

Raw sequencing reads underwent rigorous preprocessing: (1) Adapter sequences and low-quality bases (Phred score < 10 in >20% of reads) were trimmed using fastp v0.23.2 [26], (2) Reads containing >5% ambiguous nucleotides (N) were discarded. Processed reads were aligned to the *Ovis aries* reference genome (ARS-UI_Ramb_v3.0) using HISAT2 v2.2.1 [27] with default parameters. Transcript abundances were estimated as fragments per kilobase per million mapped reads (FPKM) using RSEM v1.3.3 [28]. Batch effects were assessed through principal component analysis (PCA) and Pearson correlation matrices implemented in R function princomp, and results were visualized with ggplot2 package [29]. Differential expression between breeds was determined using DEGseq v1.52.0 [30], applying thresholds of |log2(fold change)| ≥1 and Benjamini-Hochberg adjusted p-value ≤ 0.05.

Gene Ontology (GO) and KEGG pathway enrichment analyses were conducted via clusterProfiler v4.8.1 [31], with significance threshold set at adjusted *p* ≤ 0.05. Protein-protein interaction (PPI) networks were reconstructed using STRING (https://cn.string-db.org/; high-confidence score ≥ 0.7) and visualized in Cytoscape v3.10.2 [32].

### Quantitative real-time polymerase reaction (qRT-PCR)

Total RNA was isolated from full-thickness skin biopsies using the RNAeasy™ Animal RNA Isolation Kit (Beyotime, Cat No.R0026) with on-column DNase I treatment. First-strand cDNA was synthesized from 1 µg total RNA using the BeyoRT™ cDNA Synthesis Kit (Beyotime, Cat No.: D7168S) under optimized conditions: 25 °C for 10 min, 42 °C for 50 min, and 85 °C for 5 min. Quantitative PCR was performed on a LightCycler^®^ 96 System (Roche) using BeyoFast™ SYBR Green qPCR Mix (2× UDG, Cat No.:D7501) in technical triplicates. Each 20 µL reaction contained: 10 µL SYBR Green Master Mix (ROX passive reference dye included), 0.4 µM forward/reverse primers (Table S1), and 2 µL template cDNA (diluted 1:10). Relative gene expression was calculated using the 2^−ΔΔ^Ct method with three biological replicates. Glyceraldehyde-3-Phosphate Dehydrogenase (*GAPDH*) gene was selected as internal control.

### Data analysis

Experimental data was expressed as mean ± standard deviation (SD). Statistical differences between two groups were examined through two-tailed student’s t-test. One-way ANOVA was applied to check the differences among three or more groups. All data analysis were performed using Graphpad prism (version: 10.0).

## Results

### Phenotypic characteristics of hair fibers and skins in Australian white sheep and Hu sheep

**Fig. 1:**
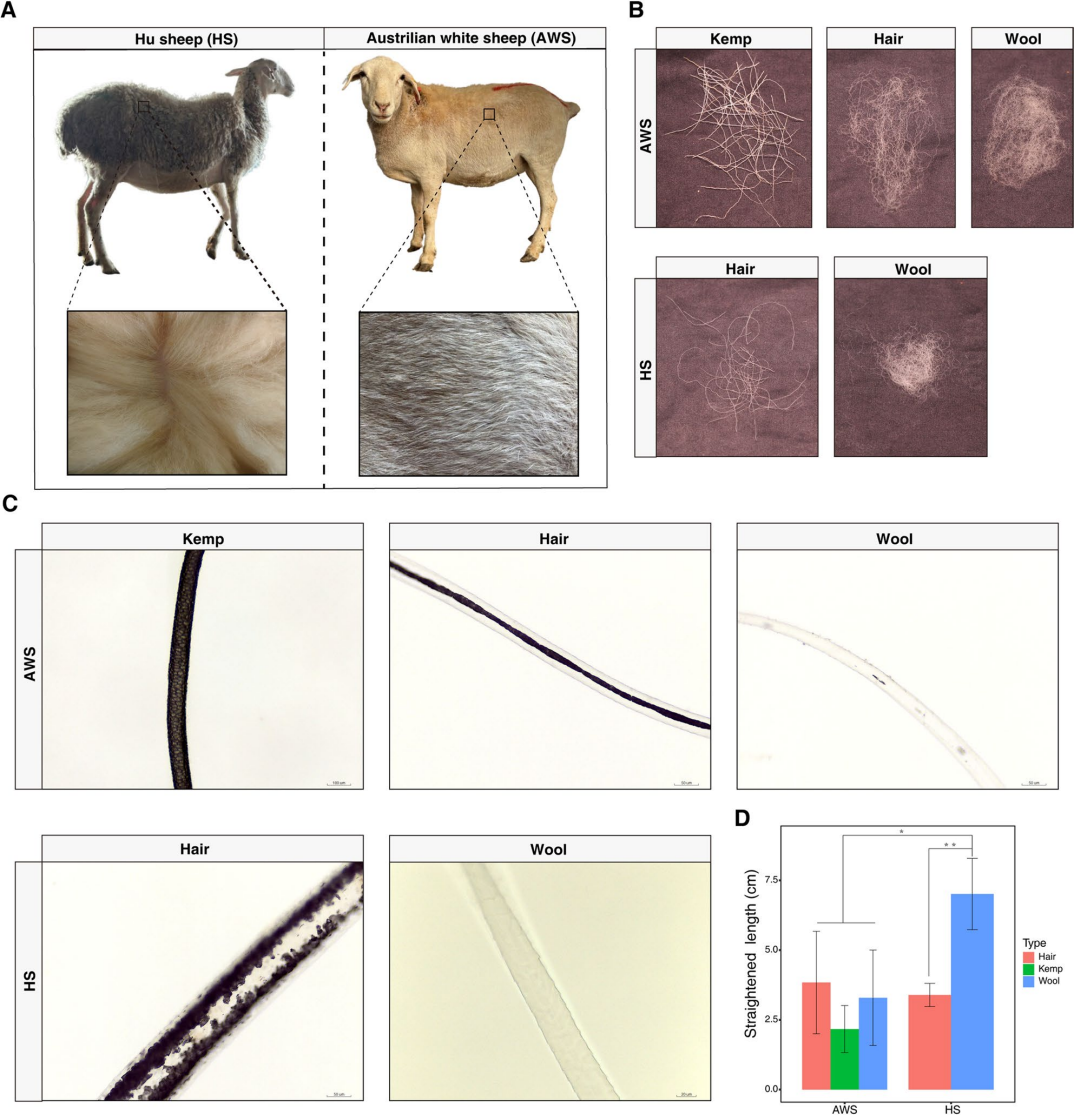
Comparative pelage morphology and fiber characteristics between hairy (AWS) and coarse-wooly (HS) sheep. **A** Representative macroscopic images of AWS (hairy phenotype) and HS (coarse-wooly phenotype), with corresponding fleece samples. **B** Gross morphology of fiber subtypes: kemp (rigid, medullated), hair (flexible, medullated), and wool (non-medullated). **C** Light micrographs of internal medullary structure. AWS hair fibers exhibit continuous cellular arrangements (top), while HS counterpart shows discontinuous medullae (middle). **D** Quantification of straightened fiber lengths (mean ± SD). **p < 0.01, *p < 0.05 by one-way ANOVA; *n* = 5 biological replicates. Scale bar: kemp, 100 μm; hair and wool from AWS, 50 μm; wool from HS, 20 μm

To elucidate the genetic mechanisms underlying pelage divergence between Australian White sheep (AWS, hairy breed) and Hu sheep (HS, coarse-woolly breed), we conducted comparative analyses of hair fiber morphology and skin histology. Macroscopic evaluation revealed striking interspecific differences: HS pelage exhibited uniformly long, fine, and lustrous fibers (Fig. 1A), whereas AWS displayed three distinct fiber types—kemp, heterotypic hair, and wool (Fig. 1B). Microscopy analysis revealed structural disparities, showing AWS kemp fibers possessed thick (> 60% diameter), opaque medullae with periodic cellular arrangements, contrasting with HS hair medullae that appeared irregular and discontinuous (Fig. 1C). While both breeds produced non-medullated wool, AWS specimens exhibited sporadic medullary remnants absent in HS (Fig. 1C). Morphometric analysis confirmed HS wool as the longest fiber type (7.0 ± 1.3 cm; *p* < 0.05), with AWS kemp being shortest (2.2 ± 0.8 cm) (Fig. 1D).

**Fig. 2:**
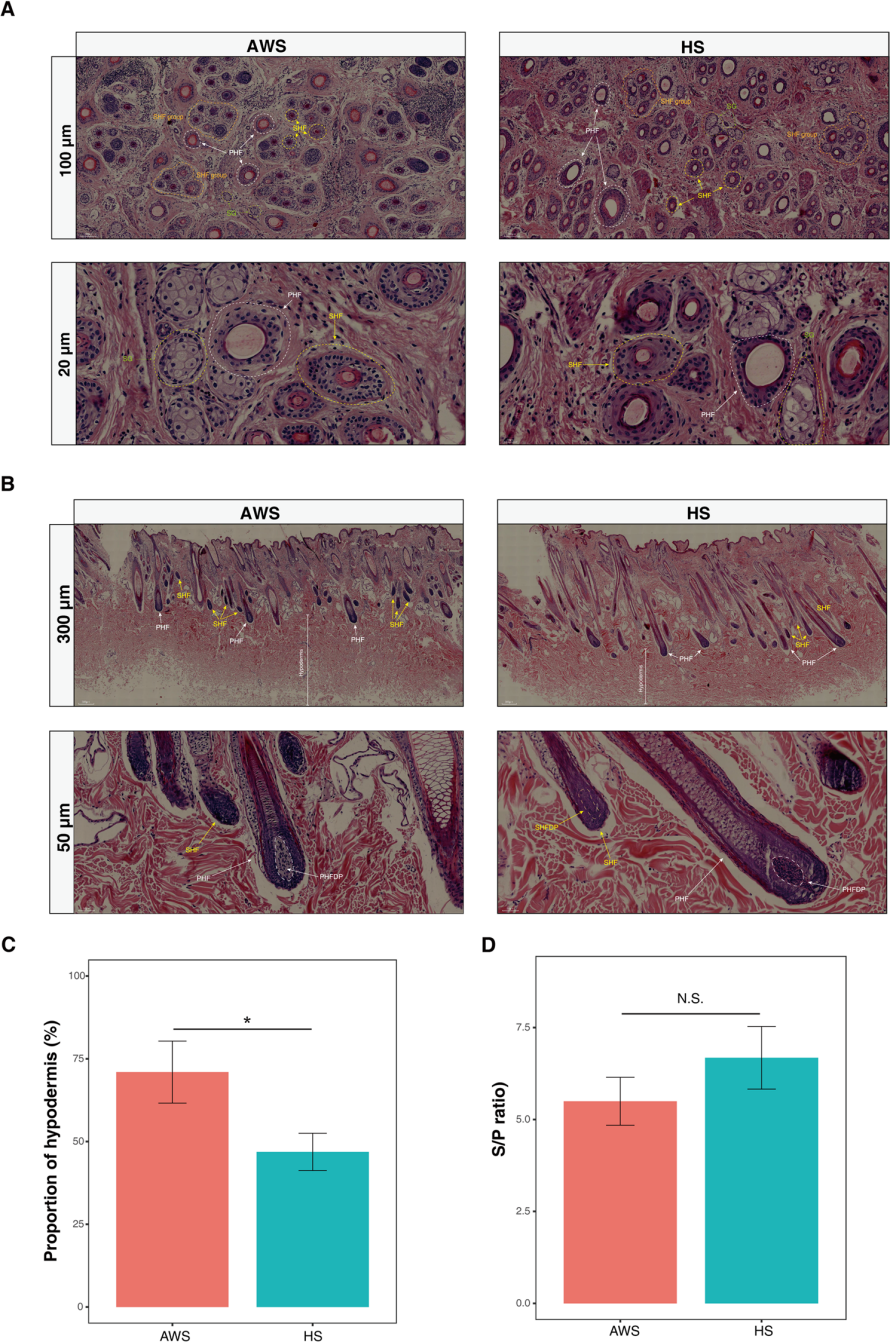
Histomorphometric analysis of integumentary architecture. **A** Transverse H&E-stained sections showing primary hair follicles (PHFs, white arrows) and clustered secondary hair follicles (SHFs, yellow arrows). Scale bar: 500 μm. **B** Longitudinal sections demonstrating PHF-SHF spatial relationships. Scale bar: 200 μm. **C** Hypodermal adipose tissue proportion relative to total skin thickness. **D** PHF: SHF ratio (S/P). Data expressed as mean ± SD; *p < 0.05 by two-tailed Student’s t-test; *n* = 5 biological replicates. NS: not significant

Histomorphological examination of transverse skin sections demonstrated conserved follicular architecture between breeds, featuring primary hair follicles (PHFs) associated with sebaceous glands and clustered secondary hair follicles (SHFs; ~5 per group) (Fig. 2A). Longitudinal analysis revealed HS dermal thickness (1,930 ± 423 μm) significantly exceeded AWS (966 ± 230 μm; *p* < 0.01), while total skin thickness remained comparable (Table S2). Proportional tissue composition differed markedly: HS skins contained 53.94 ± 4.80% dermis versus 25.69 ± 7.69% in AWS (*p* < 0.01), whereas AWS hypodermis (70.98 ± 9.37%) dominated over HS (46.87 ± 5.62%; *p* < 0.05) (Fig. 2C). The SHF to PHF ratio (S/P) showed no significant interbreed variation (AWS: 5.5 ± 1.2; HS: 6.7 ± 1.4; *p* = 0.19) (Fig. 2D). These findings establish distinct phenotypic signatures in fiber composition and skin stratification between hairy and coarse-woolly breeds.

### Screening of differentially expressed genes (DEGs) from skin tissues

**Fig. 3:**
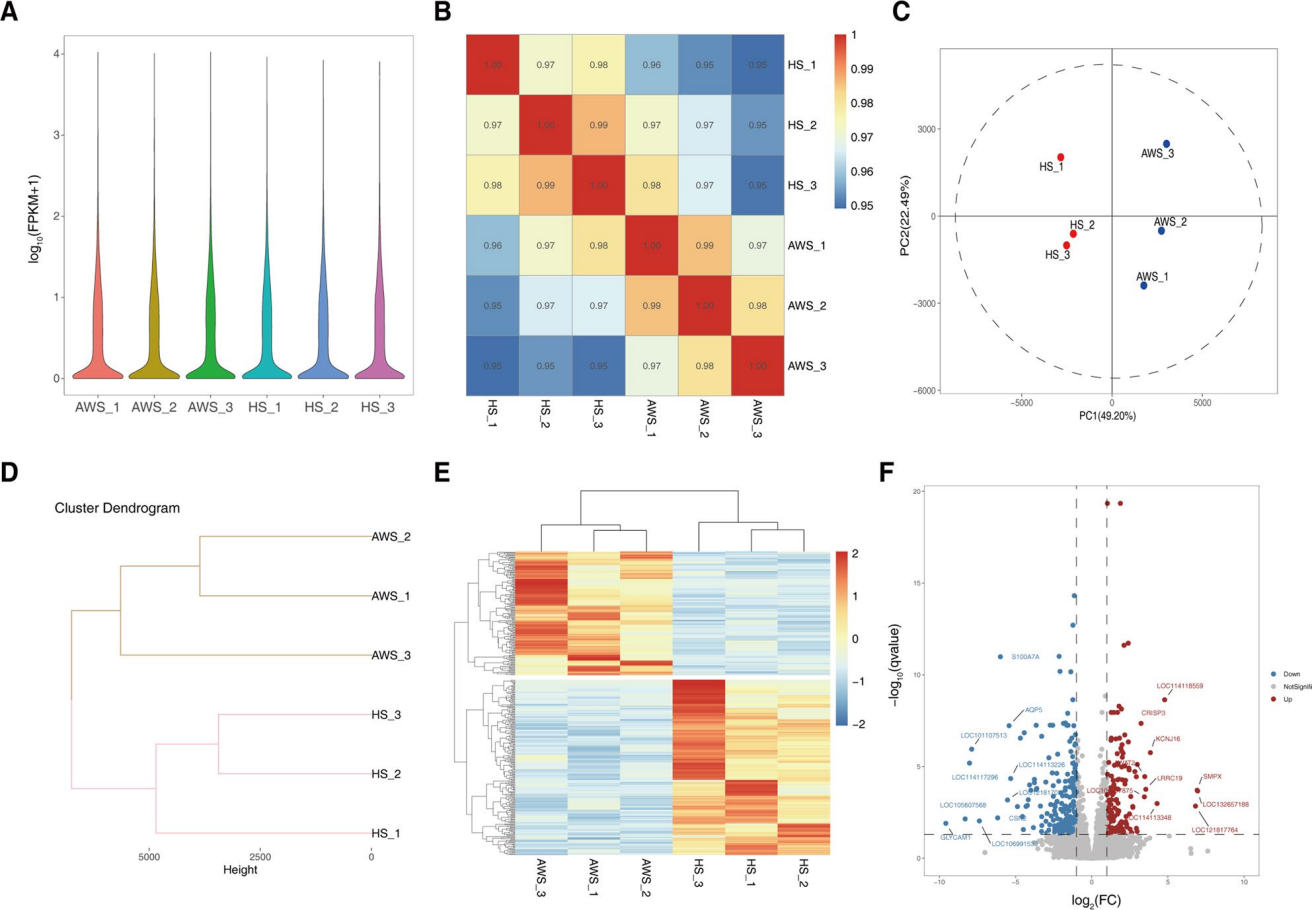
Transcriptomic landscape of AWS vs. HS skin tissues. **A** Global gene expression profiles visualized by FPKM (fragments per kilobase per million mapped reads). **B** Inter-sample Pearson’s correlation matrix. **C** Principal component analysis (PCA) plot demonstrating breed-specific clustering. **D** Hierarchical clustering dendrogram. **E** Heatmap of 370 differentially expressed genes (DEGs; |log2FC| ≥1, padj < 0.05). **F** Volcano plot highlighting DEG distribution

To screen molecular determinants underlying phenotypic divergence in pelage characteristics, transcriptome profiling was conducted on dorsal skin samples from AWS and HS. Libraries prepared from total RNA generated 45.65–94.79 million raw paired-end reads per sample, with post-quality control retention of 6.81–14.12 Gb clean data (Q20 > 98%, Q30 > 95%). Reference genome alignment (ARS-UI_Ramb_v3.0) achieved mapping rates exceeding 94.13% across all samples (detailed metrics in Table S3). Gene expression quantification using FPKM (fragments per kilobase per million mapped reads) revealed conserved global transcriptional profiles between biological replicates (Fig. 3A). Inter-sample correlation analysis demonstrated strong intra-group consistency (Pearson’s *r* = 0.97–0.99) relative to inter-breed comparisons (*r* = 0.95–0.98) (Fig. 3B). Multivariate assessment via principal component analysis (PCA) and hierarchical clustering confirmed breed-specific expression patterns of genes (Figs. 3C-D).

Differential expression analysis (|log2FC|≥1, FDR < 0.05) identified 370 DEGs, comprising 153 AWS-enriched and 217 HS-enriched transcripts (Fig. 3E-G; Table S4). Top upregulated genes in AWS included *LOC132657188* (endogenous retrovirus group K member 6 Env polyprotein-like), *SMPX* (sarcomeric protein), lipid metabolism regulators *LOC114118559* (fatty acid desaturase 2-like protein FADS2B), and *LOC114113348* (KAP20-2). Conversely, HS exhibited elevated expression of immune mediators (*GLYCAM1*, *CSF3R*) and lactogenic markers (*CSN2*), alongside structural keratin genes (*KRT25*, *DSG4*).

### Functional enrichment analysis of differentially expressed genes (DEGs) between AWS and HS

**Fig. 4:**
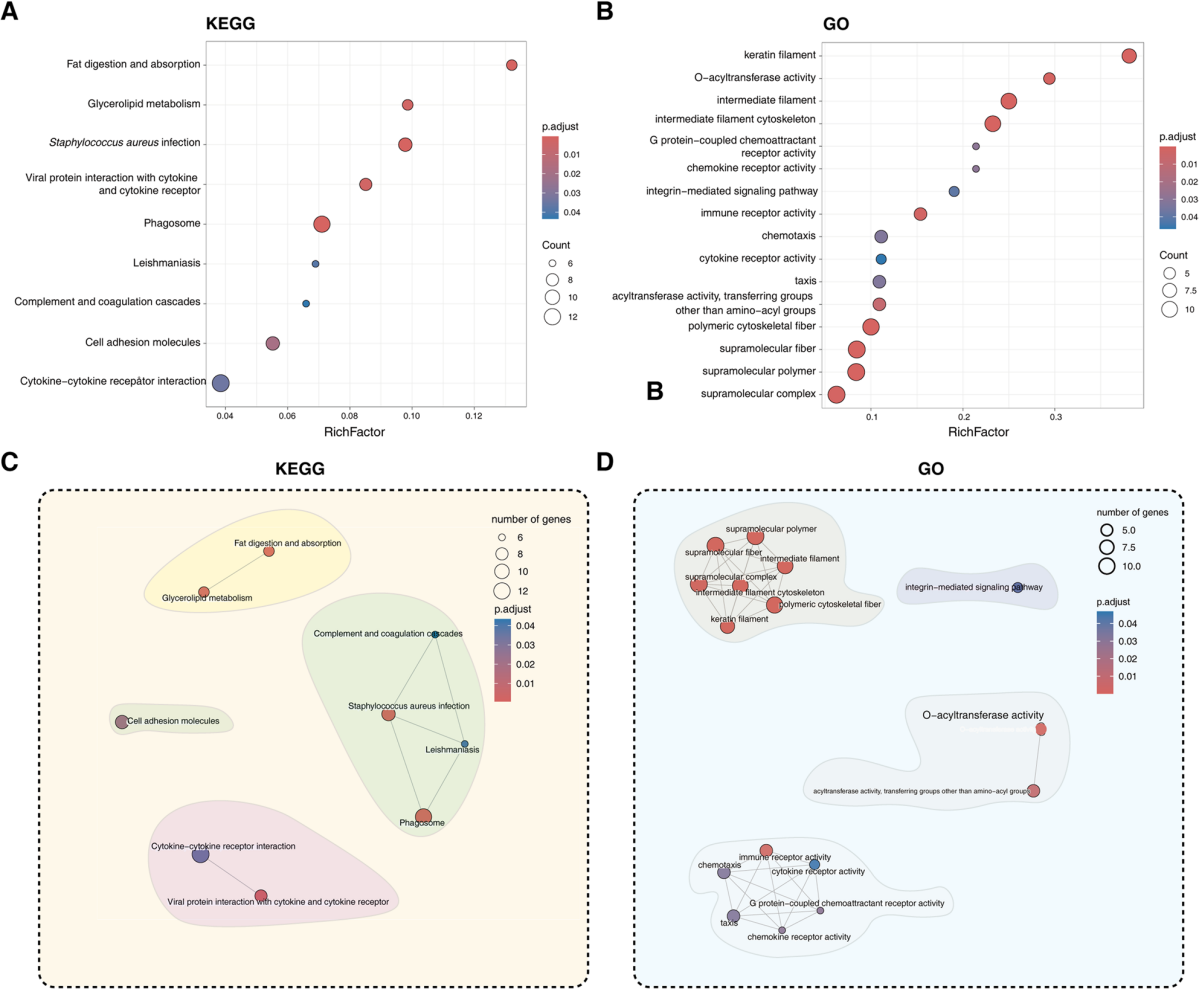
Functional enrichment analysis of DEGs between AWS and HS. **A** KEGG pathway enrichment. **B** Gene Ontology (GO) term analysis. **C** Modular representation of enriched KEGG pathways. **D** Network visualization of GO term associations. Circle size corresponds to significance level (-log_10_(padj))

To elucidate the biological significance of identified DEGs, we performed comprehensive KEGG pathway and Gene Ontology (GO) enrichment analyses. Our results revealed pronounced enrichment in lipid metabolism pathways (fat digestion and absorption: oas04975, padj = 6.6 × 10^− 4^; glycerolipid metabolism: oas00561, padj = 2.8 × 10^− 3^) and immune-related pathways (cytokine-cytokine receptor interaction: oas04060, padj = 0.034; *Staphylococcus aureus* infection: oas05150, padj = 7.1 × 10^− 6^) (Fig. 4A; Table S5). GO analysis further identified keratin filament (GO:0045095, padj = 3.8 × 10^− 8^), O − acyltransferase activity (GO:0008374, padj = 5.5 × 10^− 4^), intermediate filament (GO:0005882, padj = 5.5 × 10^− 4^), intermediate filament cytoskeleton (GO:0045111, padj = 4.0 × 10^− 8^), G protein-coupled chemoattractant receptor activity (GO:0001637, padj = 0.03), and others as predominant biological terms (Fig. 4B; Table S5).

Construction of the KEGG pathways network revealed three major sub-networks: one associated with lipid metabolism (fat digestion and absorption, glycerolipid metabolism), another primarily related to bacterial, viral infections and immune (leishmaniasis, *Staphylococcus aureus* infectionm, complement and coagulation cascades, phagosome), and the third linked to signaling molecules and interaction (cell adhesion molecules, cytokine-cytokine receptor interaction) (Fig. 4C). Similarly, network analysis of GO terms also identified three major sub-networks: one involved in lipid biosynthesis (O-acyltransferase activity, O-acyltransferase activity acyltransferase activity, transferring groups other than amino-acyl groups), another related to hair fiber composition (keratin filament, supramolecular polymer, and others), and the third associated with immune function (immune receptor activity, chemokine receptor activity, and others) (Fig. 4D). In summary, on the one hand, these results confirm the differences in fleece composition between hairy sheep and wooly sheep as observed in the aforementioned results, and this is also consistent with the difference in subcutaneous adipose tissue thickness between the two sheep breeds. On the other hand, they further suggest that the immune system may be involved in the phenotypic differences.

### Upregulated DEGs in AWS predominantly associate with lipid metabolic processes​

**Fig. 5:**
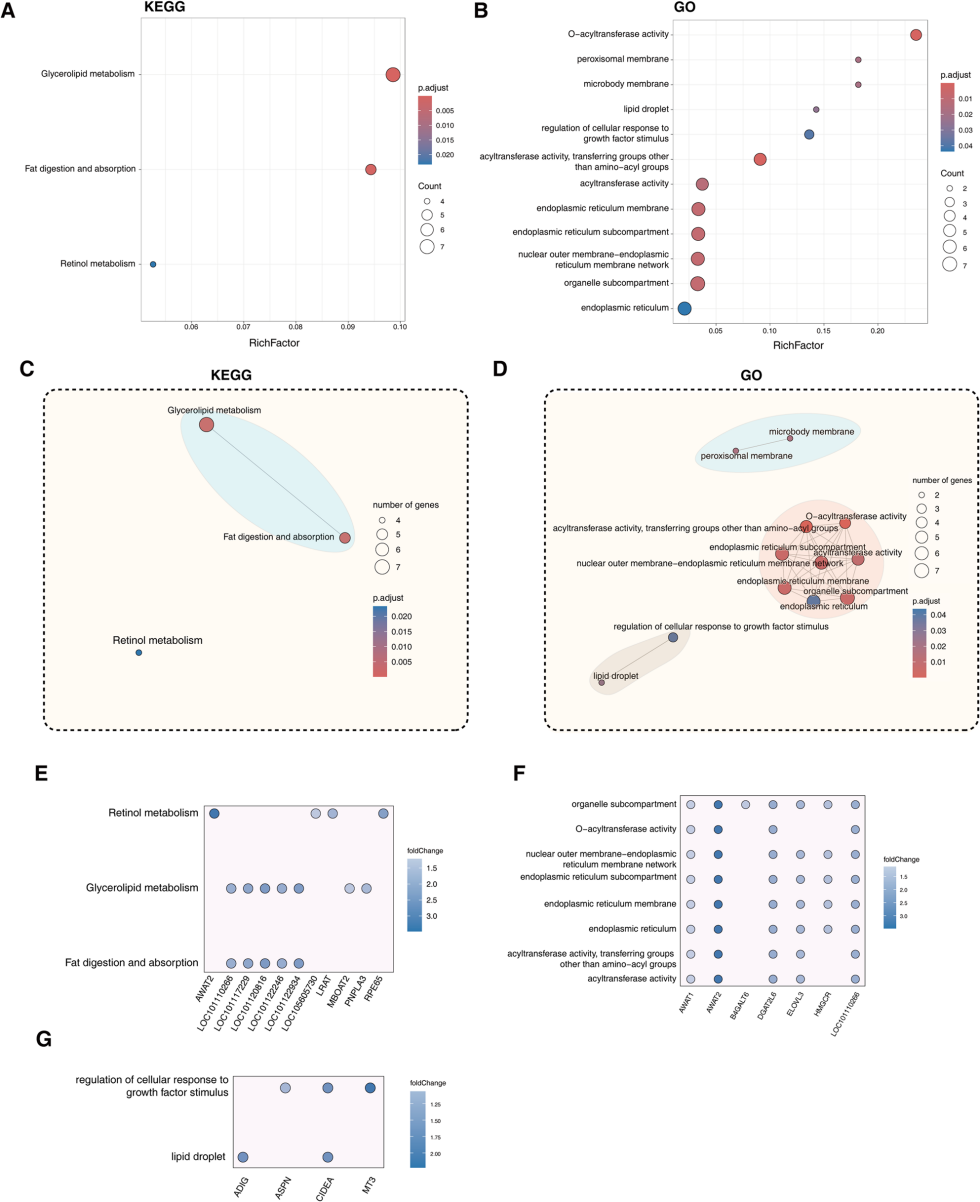
Functional enrichment analysis of DEGs upregulated in AWS. **A** KEGG pathways and (**B**) GO terms associated with AWS-upregulated genes. **C**, **D** Functional module interactions. **E**-**G** Gene-pathway/GO term association networks

To delineate the biological significance of AWS-upregulated DEGs, similar functional enrichment analysis protocol were performed. KEGG analysis identified three core metabolic pathways: glycerolipid metabolism (oas00561, padj = 7.5 × 10⁻^6^), fat digestion/absorption (oas04975, padj = 4.0 × 10⁻^4^), and retinol metabolism (oas00830, padj = 0.02). GO analysis also highlighted the enrichment of lipid metabolism-related terms including O-acyltransferase activity (GO: 0008374, padj = 9.0 × 10⁻^5^), the regulation of cellular response to growth factor stimulus (GO:0090287, padj = 9.0 × 10⁻^5^), and cellular components such as lipid droplets (GO:0005811, padj = 0.03), and others (Fig. 5A-B; Table S5). Network analysis of KEGG pathways and GO terms also identified functionally highly connected terms, along with their corresponding sub-networks (Fig. 5C-D). Meanwhile, DEGs associated with above functional terms have also been presented. For example, six acyltransferase-encoding genes—including *MBOAT2*, *PNPLA3*, and four 2-acylglycerol O-acyltransferase isoforms (*LOC101110266*, *LOC101110229*, *LOC101120816*, *LOC101122246*)—collectively participate in the glycerolipid metabolism pathway. At the same time, the four isoforms also participate in the fat digestion and absorption pathway (Fig. 5E). Additionally, a set of genes—including *DGAT2L6*, *ELOVL3*,* ADIG*, and *CIDEA* [33, 34]—that play important roles in adipocyte development, fat deposition, and metabolism were also found to be associated with specific signaling pathways (Fig. 5F-G).

We have also provided a manually curated list of genes associated with lipid metabolism (Table 2), specifically to address the insufficiency of gene functional annotation. For instance, *FOXO6* (log₂FC = 1.74, padj = 3.17 × 10⁻^3^) enhanced lipid retention through PPARγ co-activation [35], while *SFRP2* (log₂FC = 1.23, padj = 3.16 × 10⁻^3^) potentiated adipogenic potential of stem cells through inhibiting Wnt/β-catenin signaling [36]. Overall, the specifically high expression of these lipid metabolism-related genes in AWS further confirms the excessive subcutaneous fat deposition.

**Table 2:** Specific upregulation of lipid metabolism related DEGs in AWS

Gene symbol	Gene description	log_2_FC	Adjusted *p*-value
*LOC114118559*	fatty acid desaturase 2-like protein FADS2B	4.79	2.30E-09
*AWAT2*	acyl-CoA wax alcohol acyltransferase 2	3.48	3.56E-05
*LOC101117875*	fatty acid desaturase 2-like protein FADS2B	3.46	4.52E-04
*LOC101120816*	2-acylglycerol O-acyltransferase 2-like	2.46	1.54E-05
*LOC101122934*	2-acylglycerol O-acyltransferase 2-like	2.44	1.22E-05
*MT3*	metallothionein 3	2.22	5.70E-05
*SOST*	sclerostin	2.20	1.18E-02
*LOC101117229*	2-acylglycerol O-acyltransferase 3	2.12	2.48E-12
*ELOVL4*	ELOVL fatty acid elongase 4	2.01	3.15E-06
*LOC101116336*	liver carboxylesterase-like	1.99	6.49E-07
*LOC101122246*	2-acylglycerol O-acyltransferase 3	1.96	7.36E-09
*DGAT2L6*	diacylglycerol O-acyltransferase 2 like 6	1.95	1.02E-02
*NNAT*	neuronatin	1.94	1.31E-02
*LOC101110266*	2-acylglycerol O-acyltransferase 2-like	1.92	1.02E-06
*SNED1*	sushi, nidogen and EGF like domains 1	1.88	4.53E-20
*FA2H*	fatty acid 2-hydroxylase	1.87	4.31E-02
*ENPEP*	glutamyl aminopeptidase	1.87	2.10E-02
*FAR2*	fatty acyl-CoA reductase 2	1.83	2.84E-07
*SOAT1*	sterol O-acyltransferase 1	1.79	5.16E-09
*SERPINE1*	serpin family E member 1	1.78	1.74E-02
*FOXO6*	forkhead box O6	1.74	3.17E-03
*LRAT*	lecithin retinol acyltransferase	1.74	1.72E-04
*CIDEA*	cell death inducing DFFA like effector a	1.73	1.01E-03
*ADIG*	adipogenin	1.70	2.47E-03
*DIO1*	iodothyronine deiodinase 1	1.70	3.94E-03
*ELOVL3*	ELOVL fatty acid elongase 3	1.69	7.99E-06
*ERICH4*	glutamate rich 4	1.63	3.14E-02
*PNPLA3*	patatin like phospholipase domain containing 3	1.62	6.37E-04
*GNAL*	G protein subunit alpha L	1.59	3.14E-07
*HMGCR*	3-hydroxy-3-methylglutaryl-CoA reductase	1.46	1.13E-08
*LOC101120029*	phospholipase A and acyltransferase 3-like	1.37	3.45E-02
*LOC114108774*	sterol carrier protein 2-like	1.29	6.42E-04
*MBOAT2*	membrane bound O-acyltransferase domain containing 2	1.27	1.16E-03
*SFRP2*	secreted frizzled related protein 2	1.23	3.16E-03
*CERS4*	ceramide synthase 4	1.23	2.39E-03
*LOC101119041*	ultra-long-chain fatty acid omega-hydroxylase	1.17	2.57E-03
*APLNR*	apelin receptor	1.15	2.07E-03
*AWAT1*	acyl-CoA wax alcohol acyltransferase 1	1.13	4.35E-02
*CRAT*	carnitine O-acetyltransferase	1.13	3.41E-03

### Overexpressed DEGs in HS are primarily associated with Immunomodulation and hair fiber composition

**Fig. 6:**
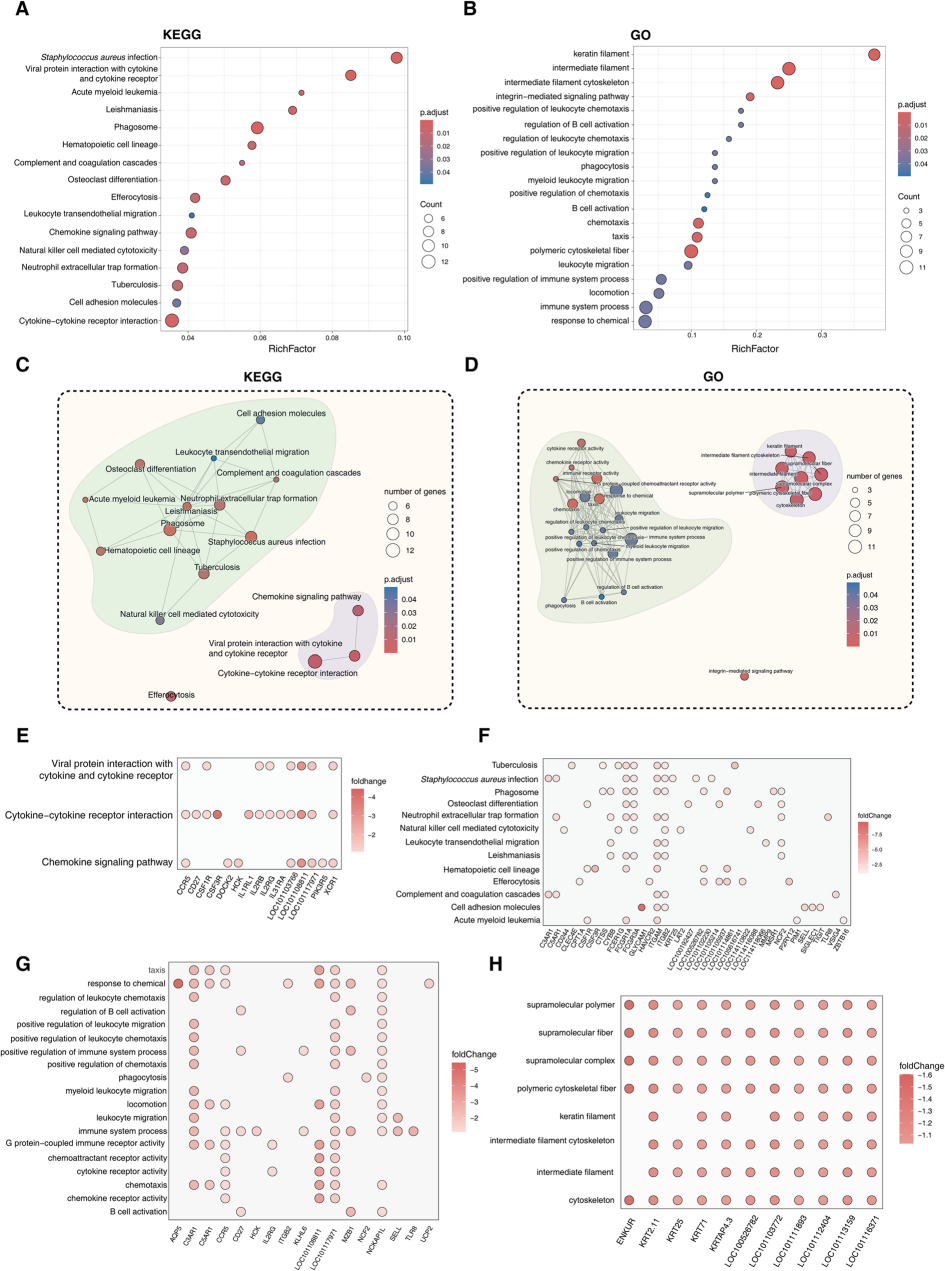
Functional enrichment analysis of DEGs downregulated in AWS. **A**, **B** Enriched KEGG pathways and GO terms. **C**, **D** Functional modules of enriched pathways and GO terms. **E**-**H** Gene-term interaction networks

KEGG and GO enrichment analyses, along with subsequent network analysis, of HS-specifically expressed genes demonstrate that these DEGs are primarily associated with immunomodulation and hair composition (Fig. 6A-D). For instance, in the GO network analysis, a large number of GO terms related to immunomodulation—such as regulation of B cell activation—form an independent subnetwork, whereas GO terms associated with animal hair fiber structure (e.g., intermediate filament) form another sub-network. The correspondence between key functional terms and their associated genes has also been presented (Fig. 6E-H). For instance, a group of KRT genes (*KRT25*, *KRT71*, *KRT2.11*) and KAP genes (*KRTAP4.3*) are involved in keratin filament and other GO terms related to hair formation.

Importantly, a rich list of hair fiber trait-associated DEGs was provided in Table 3. Notably, *LOC114113348* (keratin-associated protein 20 − 2, log_2_FC = 4.29), a key determinant of fiber curvature in ovine species [14], showed maximal upregulation in AWS. Despite this specificity, the expression abundances of the majority of relevant DEGs are down-regulated in HS. Meanwhile, the top 10 membrane receptors associated with immune cell function regulation have also been listed (Table 4). For instance, *CSF3R*, the top-ranked gene (log_2_FC= −4.43), was identified as a surface marker for myeloid-derived suppressor cells (MDSCs), a type of cells with immunosuppressive function [37]. Overall, these hair-related genes may serve as key candidate genes contributing to differences in hair composition between AWS and HS, while the immune system may act as a key pathway underlying differences in coat phenotype between the two groups.

**Table 3:** Expression levels of pelage phenotype-related DEGs in Hu sheep (HS) and Australian white sheep (AWS)

Gene symbol	Gene description	log_2_FC	Adjusted *p*-value
*LOC114113348*	keratin-associated protein 20 − 2	4.29	1.05E-03
*LOC101111178*	keratin, type II cytoskeletal 6 A-like	2.17	9.44E-06
*LOC114113269*	keratin-associated protein 21 − 1	1.36	1.88E-04
*EDA2R*	ectodysplasin A2 receptor	1.23	2.44E-03
*LOC101115634*	keratin-associated protein 9 − 2	−1.73	6.44E-05
*LOC101114537*	keratin-associated protein 4–9-like	−1.55	5.68E-08
*LOC114112036*	keratin-associated protein 9-2-like	−1.35	7.07E-05
*KRT2.11*	hair keratin type II intermediate filament	−1.28	4.39E-05
*LOC101103772*	keratin-associated protein 11 − 1	−1.24	2.30E-09
*KRT33A*	keratin 33 A	−1.23	1.99E-13
*LOC101113159*	keratin, high-sulfur matrix protein, B2D	−1.22	9.85E-05
*LOC101120969*	trichohyalin	−1.20	8.30E-05
*LOC100526782*	keratin 33B	−1.17	5.97E-08
*LOC101116371*	keratin-associated protein 13 − 1	−1.16	2.37E-04
*LOC106991427*	keratin-associated protein 4–9-like	−1.13	2.46E-02
*LOC101112404*	keratin-associated protein 3 − 2	−1.10	6.84E-07
*KRTAP4.3*	keratin associated protein 4.3	−1.10	7.99E-06
*KRT71*	keratin 71	−1.10	1.97E-05
*LOC114116843*	keratin-associated protein 9-2-like	−1.09	1.22E-05
*LOC100526784*	keratin 81	−1.07	5.81E-07
*KRT25*	keratin 25	−1.05	5.56E-05
*LOC114116854*	keratin, high-sulfur matrix protein, IIIA3A	−1.04	3.62E-05
*LOC101111893*	keratin, high sulfur matrix protein, IIIB3d	−1.03	6.44E-05
*LOC114116849*	keratin-associated protein 9-2-like	−1.02	5.55E-03

Hu sheep (HS) were set as control group

**Table 4:** Expression levels of top 10 immune cell related cell surface markers

Gene symbol	Gene description	log_2_FC	Adjusted *p*-value
*CSF3R*	colony stimulating factor 3 receptor	−4.43	1.45E-07
*LOC101108811*	C-X-C chemokine receptor type 2	−3.36	1.44E-03
*LOC114118066*	leukocyte immunoglobulin-like receptor subfamily A member 5	−3.01	9.72E-03
*TLR8*	toll like receptor 8	−2.72	5.62E-08
*C3AR1*	complement C3a receptor 1	−2.52	4.23E-04
*C5AR1*	complement C5a receptor 1	−1.93	1.26E-02
*MSR1*	macrophage scavenger receptor 1	−2.36	4.18E-03
*LOC101117971*	C-C chemokine receptor type 1	−1.70	7.67E-03
*FOLR2*	folate receptor beta	−1.66	1.43E-02
*GLP2R*	glucagon like peptide 2 receptor	−1.60	2.12E-03

### Protein-protein interaction network analysis of DEGs​

**Fig. 7:**
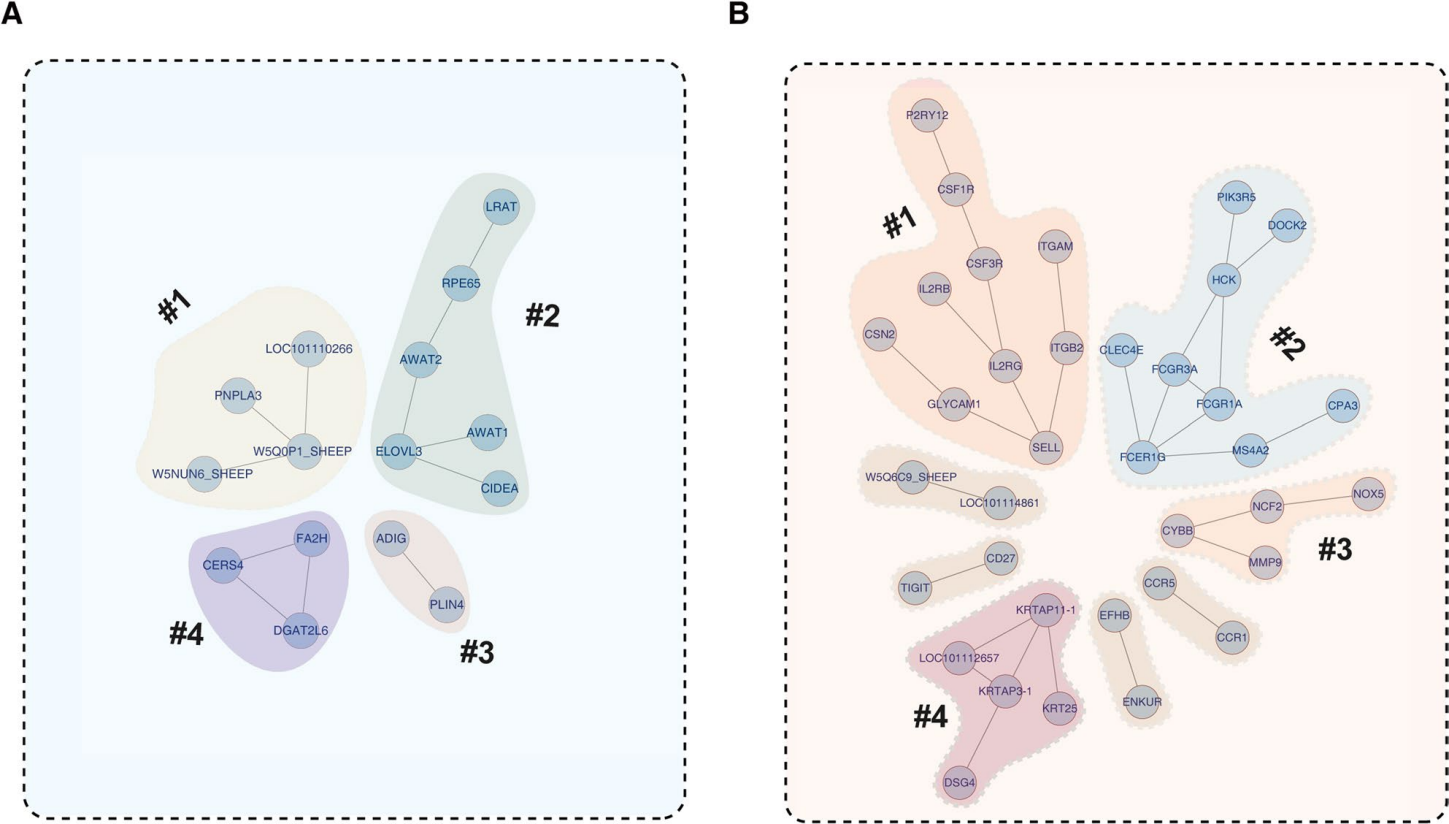
Protein-protein interaction (PPI) networks. **A** AWS-upregulated DEG cluster centered on lipid metabolism. **B** HS-upregulated DEG modules: immune regulation and keratinization. Interaction confidence score ≥ 0.7

To elucidate functional interdependencies among DEGs, we constructed protein-protein interaction (PPI) networks. Four independent sub-networks are formed by the upregulated genes in AWS (Fig. 7A), suggesting that genes within each network may participate in distinct aspects of lipid metabolism through a coordinated manner. For instance, genes in sub-network #1 primarily perform acyltransferase functions. Genes associated with HS are also organized into several sub-networks: one of these networks includes KRTAP11-1 and other related proteins, while the remaining sub-networks are primarily associated with the immune system. However, these networks are not interconnected with one another, suggesting that they may be involved in immune regulation through distinct functioning processes. For instance, genes in sub-network #2 are primarily associated with innate immunity. These results facilitate a deeper understanding of the roles of lipid metabolism and immunity in influencing coat phenotypes in the two sheep breeds (i.e., AWS and HS) from the perspectives of gene function and biological processes.

### Validation of transcriptomic data by qRT-PCR​

Technical validation of RNA-seq findings was performed through quantitative reverse transcription PCR (qRT-PCR) on 6 randomly selected DEGs (3 upregulated in AWS, 3 in HS). Similar expression trends were observed between sequencing and qRT-PCR results, with all genes recapitulating their directional expression patterns (Table S5). This high reproducibility confirms the robustness of our transcriptomic profiling methodology and reinforces the biological validity of identified DEGs.

## Discussion

Herein, we explored potential genetic mechanisms underlying pelage divergence between hairy and coarse-woolly sheep by integrating comparative histomorphological characterization of hair fibers and skin with high-throughput transcriptomic profiling of skin tissues from Australian White sheep (AWS, a representative hairy breed) and Hu sheep (HS, a typical Chinese coarse-woolly breed). Our multi-dimensional analysis identified striking phenotypic differences between the two breeds, including key traits of fleece architecture and marked variation in subcutaneous adiposity (Figs. 1 and 2). Transcriptomic analysis further identified functional genes and biological processes that are potentially closely associated with these phenotypic differences (Figs. 3, 4, 5, 6 and 7). These findings not only provide reliable clues for the subsequent identification of candidate genes applicable for marker-assisted breeding, but also offer potential research directions for deepening our understanding of inter-breed pelage phenotypic differences in sheep.

The results of hair fiber type analysis for the two sheep breeds are consistent with prior reports [4, 5], and further align with the pattern of pelage composition changes during sheep domestication—specifically the transition from wild to domestic sheep [3, 38]. Hair fiber lengths also align with the patterns of pelage development: specifically, both hair and wool fibers of hairy sheep exhibit seasonal growth and shedding, whereas the fleece of woolly sheep undergoes continuous growth [1]. The range of S/P ratios and their differences between the two sheep breeds (AWS, 5.5; HS, 6.7) are highly consistent with the fact that long-term artificial selection for enhanced wool production performance resulted a gradual increase in the proportion of secondary HFs [2]. Notably, AWS exhibit substantially greater subcutaneous adipose tissue thickness compared to HS (Fig. 2). This observation corroborates earlier reports that hairy sheep breeds (e.g., Dorper) possess greater subcutaneous adipogenic capacity than wooly types (e.g., Merino) [23, 24]. More importantly, the inverse relationship between subcutaneous adiposity and hair fiber length aligns with clinical observations in patients with androgenetic alopecia, who display increased frontal fat pad adiposity compared to healthy controls [39]. This cross-species consistency not only validates the link between adiposity and follicular traits but also highlights a potential conserved role of adipocytes in inhibiting folliculogenesis—an insight that advances our understanding of the shared regulatory mechanisms underlying hair/wool growth across mammals.

The observed subcutaneous adiposity disparity between AWS and HS was corroborated at molecular level through differential expression analysis. Transcriptomic profiling revealed significant enrichment (padj < 0.05) of lipid metabolism pathways in AWS, particularly involving triacylglycerol biosynthesis regulators (Figs. 3 and 4; Table 2). Key enzymes in neutral lipid synthesis – including monoacylglycerol O-acyltransferases (*LOC101120816*, *LOC101122934*) and diacylglycerol O-acyltransferase *DGAT2L6* – showed ≥ 2-fold elevated expression in AWS skins (padj < 0.05). This metabolic predisposition was further supported by upregulated adipogenic regulators: *ADIG* (log₂FC = 1.70) promoting lipid droplet biogenesis [33], *CIDEA* (log₂FC = 1.72) enhancing lipid storage [34], and *FOXO6* (log₂FC = 1.73) mediating adipocyte differentiation [35]. Paradoxically, MT3 (log₂FC = 2.22) – a zinc metallothionein inhibiting preadipocyte commitment [40] – was concurrently elevated, suggesting complex regulatory interplay in adipose homeostasis. The adipogenic transcriptomic landscape provides mechanistic insights into the inverse correlation between subcutaneous fat deposition (70.98 ± 9.37% in AWS vs. 46.87 ± 5.62% in HS, *p* < 0.05) and hair length phenotypes. Mature adipocytes secrete BMP2, a potent inhibitor of hair follicle stem cell activation [39], while co-culture experiments demonstrate subcutaneous fat reduces follicular cell proliferation by 86% [41]. Furthermore, AWS exhibited elevated expressions of fatty acid modification enzymes: FADS2 paralogs (*LOC114118559*, log₂FC = 4.79; *LOC101117875*, log₂FC = 3.46) and ELOVL3/4 elongases (log₂FC = 1.69/2.01) catalyzing LC-PUFA biosynthesis from C18 precursors [42]. Although arachidonic acid (20:4n-6) stimulates human hair growth via FGF10 upregulation [43], n-3 PUFAs like DPA (22:5n-3) induce murine alopecia through macrophage-mediated inflammatory cascades [44]. This dichotomy suggests adipose-mediated PUFA balance may differentially regulate follicular activity across species.

Our transcriptomic analysis revealed significant differential expression of keratin (KRT) and keratin-associated protein (KAP) genes between hairy and coarse-woolly sheep, highlighting their critical roles in determining hair fiber characteristics. KRTs form the intermediate filament backbone while KAPs constitute the matrix scaffolding, collectively governing key physical properties including tensile strength, elasticity, and curvature [45]. Notably, *KAP20-2* (*LOC114113348*) exhibited 16.3-fold higher expression (log_2_FC = 4.29, padj = 1.05 × 10^− 3^) in AWS, consistent with its established association with wool/cashmere crimp in ovine and caprine populations [14, 46]. This molecular signature correlates with the distinctive straight kemp fibers (AWS pelage) that confer mechanical resilience against environmental abrasion. The molecular repertoire further included *KRT71* (log_2_FC=−1.10), a known regulator of hair follicle curvature across species [13, 47, 48], and *EDA2R* (log_2_FC = 1.23), an X-linked receptor inducing premature termination of hair growth through driving follicular cell apoptosis [49]. Our findings align with human study where a non-synonymous SNP mutation (rs1385699, *p* = 3 × 10^− 9^) of *EDA2R* is strongly associate with androgenetic alopecia [50], suggesting evolutionary conservation of this pathway in animal pelage development. In addition, genetic variations in *LOC101111178* (*KRT6A-like*), *LOC114113269* (*KAP21-1*), *LOC114112036* (*KAP9-2-like*), *LOC101120969* (trichohyalin), and *KRT71* are associated with key hair fiber traits (yield, curvature) across sheep, goats, and other mammals [15, 17, 48, 51–54].

Intriguingly, transcriptomic analysis revealed significant enrichment of immune-related differentially expressed genes (DEGs) and functional pathways in the skin tissues of coarse-woolly sheep (Fig. 6), highlighting a previously underappreciated role of immune regulation in shaping ovine pelage phenotypes. This finding aligns with clinical observations where immunosuppressive agents (cyclosporin A, FK506) enhance hair growth [55, 56], whereas pro-inflammatory cytokines (IL-1α, TNF-α, IFN-γ) inhibit follicular elongation [57, 58]. The immune privilege status of anagen-phase HFs [59], whose collapse underlies autoimmune alopecia areata (AA) via CD8^+^ T cell infiltration [60, 61], suggests a conserved mechanism wherein localized immunosuppression may facilitate sustained fiber growth in coarse-woolly breeds. Our data support this hypothesis through two lines of evidence: (1) immunosuppressive factor upregulation: *CSN2* (β-casein) inhibits lymphocytes expansion and reduces IFN-γ production by 35–44% in splenocytes [62], and *LOC101102413* (haptoglobin) decreases TNF-α secretion in monocytes [63], and (2) immune cell receptor modulation: *CSF3R*^+^ myeloid-derived suppressor cells inhibits *CD4*^+^*CD8*^+^ T cell proliferation [37] and NK cell cytotoxicity [64], and *CXCR2*^+^ neutrophils restrain *CD8*^+^ T cytotoxicity in tumor to facilitate immune invasion [65]. Notably, coarse-woolly sheep exhibited coordinated expression of immunoregulatory receptors (*IL2RB*/*IL2RG*, *ITGAM*/*ITGB2*, *LILRA5*/*LOC114118066*) that collectively establish an immunosuppressive microenvironment [66–68] (Table 4). However, skins are composed of multiple immunocyte types, including Langerhans cells, macrophages, dendritic cells, lymphocytes, monocytes, and others [69]. In future, single-cell RNA sequencing could resolve cell-type-specific contributions to follicular immune privilege maintenance and pelage variations in sheep.

Protein-protein interaction (PPI) analysis also facilitates an in-depth understanding of gene function and the functional coordination among genes (Fig. 7). Notably, KRTAP11-1 — a structural protein enriched in ground hairs versus guard hairs [70] — emerged as a strong candidate regulator of fiber curvature, in which the critical roles of its interaction partners KRT25 and DSG4 are well characterized [15, 71]. Furthermore, *SELL* has been reported to be a key gene involved in the regulation of leukocyte immune function, and SELL-positive Treg cells exhibit superior immunosuppressive capacity [72]. This is also consistent with the interaction between SELL and genes such as *IL2RG* and *ITGB2*, which may collectively maintain the state of immune privilege within HF.

Several limitations remain in the current study, including the inherent seasonal growth pattern of wool in hairy sheep and the inability of transcriptome analysis conducted at the bulk skin tissue level to sufficiently account for variations in traits among specific hair types. Moving forward, two lines of inquiry will serve as prioritized research directions: first, systematically investigating the dynamic variation patterns of sheep coats and skin tissues across the entire annual cycle; second, utilizing single-cell omics, proteomics, and other complementary omics technologies to further delineate the key genes and their underlying molecular mechanisms that govern pleage composition and variation—findings that could provide a theoretical foundation for modern sheep breeding.

## Conclusion

This integrated analysis delineates subcutaneous adipogenesis and immunomodulation as potential regulators of pelage divergence between hairy and coarse-wooly sheep. Elevated lipid metabolism gene expression (e.g., *DGAT2L6*, *CIDEA*, *ADIG*) in hairy breeds correlates with robust hypodermal adiposity, inversely associated with reduced hair fiber length, potentially mediated through adipokines-mediated follicular growth inhibition. Enrichment of immune genes and pathways in the skins of coarse-wooly sheep indicates that the immune system is a key factor influencing pelage traits, potentially achieved by maintaining the immune-privileged state of HF. Several DEGs, such as *KAP20-2* and *EDA2R*, may serve as key candidates that underpin phenotypic differences in sheep. Taken together, these findings provide novel insights into the molecular mechanisms underlying variations in sheep coat composition and offer reliable candidate genes for designing novel sheep breeds with enhanced environmental adaptability.

## Supplementary Information

Supplementary Material 1: Table S1. Primer sequences.

Supplementary Material 2: Table S2. Thickness of skin tissue and related components.

Supplementary Material 3: Table S3. Information of sequencing libraries.

Supplementary Material 4: Table S4. Identification of DEGs.

Supplementary Material 5: Table S5. Functional enrichment analysis of DEGs.

Supplementary Material 6: Table S6. QRT-PCR validation of RNA-seq results.

## Data Availability

All sequencing data generated this study are publicly available in the China National Center for Bioinformation database as BioProject No. PRJCA031884 and GSA NO. CRA020117.

## Abbreviations

ADIG: Adipogenin
CIDEA: Cell death inducing dffa like effector a
CLEC4E: C-type lectin domain family 4 member e
CPA3: Carboxypeptidase A3
CSF3R: Colony stimulating factor 3 receptor
CSN2: Casein beta
CXCR2: C-X-C motif chemokine receptor 2
DEGs: Differentially expressed genes
DGAT2L6: Diacylglycerol O-acyltransferase 2 like 6
DSG4: Desmoglein 4
EDA2R: Ectodysplasin A2 receptor
ELOVL3: ELOVL fatty acid elongase 3
FADS2: Fatty acid desaturase 2
FCER1G: Fc epsilon receptor Ig
FCGR1A: Fc gamma receptor Ia
FCGR3A: Fc gamma receptor IIIa
FGF5: Fibroblast growth factor 5
FGF10: Fibroblast growth factor 10
FOXO6: Forkhead box o6
FOXQ1: Forkhead box q1
FPKM: Per kilobase of exon per million mapped fragments
GAPDH: Glyceraldehyde-3-phosphate dehydrogenase
GLYCAM1: Glycosylation dependent cell adhesion molecule 1
GWAS: Genome-wide association study
GPCR: G protein-coupled receptor
HF: Hair Follicle
IFN-γ: Interferon gamma
IL-1A: Interleukin 1 alpha
IL2RB: Interleukin 2 receptor subunit beta
IL2RG: Interleukin 2 receptor subunit gamma
IRF2BP2: Interferon regulatory factor 2 binding protein 2
ITGAM: Integrin subunit alpha M
ITGB2: Integrin subunit beta 2
KAP: Keratin associated protein
KRT: Keratin
LC-PUFA: Long-chain polyunsaturated fatty acids
MS4A2: Membrane spanning 4-domains a2
MT3: Metallothionein 3
NNAT: Neuronatin
PIK3R5: Phosphoinositide-3-kinase regulatory subunit 5
SELL: Selectin L
SFRP2: Secreted frizzled related protein 2
SNPs: Single-nucleotide polymorphism
SOST: Sclerostin
TARBP1: TAR (HIV-1) RNA binding protein 1
TCHH: Trichohyalin
TNF-α: Tumor necrosis factor alpha
